# Antiretroviral therapy (ART) adherence and correlates to non-adherence among people on ART in Estonia

**DOI:** 10.1186/1742-4690-9-S1-P80

**Published:** 2012-05-25

**Authors:** Anneli Uuskula, Kaja-Triin Laisaar, Mait Raag, K Rivet Amico, Anjali Sharma, Jack DeHovitz

**Affiliations:** 1 Department of Public Health, University of Tartu, Tartu, Estonia; 2 Center for Health Intervention and Prevention, University of Connecticut, Storrs, CT, USA; 3 Division of Infectious Diseases, State University of New York Downstate Medical Center, Brooklyn, NY USA

## Introduction

There is limited data on antiretroviral therapy (ART) adherence among patients in Eastern Europe, despite the high incidence of HIV infection and the growing number of HIV-infected individuals who are being prescribed ART. We conducted a study to measure rates of adherence to ART and factors associated with non-adherence among patients receiving care at an outpatient HIV clinic in Estonia.

## Materials and methods

The study was based on cross-sectional data from a convenience sample of 161 patients receiving outpatient HIV care. Data were obtained via interviewer administered surveys and data abstraction from clinical records. Adherence was measured from 3-day patient self-report.

## Results

Among the 161 participants (mean age 33 and 55% male / 45% female), two thirds (64%) had been infected with HIV through intravenous drug use. Most (74%) were co-infected with hepatitis C (HCV). Perfect adherence over the last 3-days was commonly reported [87% (95% CI 80 − 92%)], with non-perfect adherence associated with greater concerns about the potential negative consequences of taking ART [AOR 5.8 (95% CI 1.3 − 45.7)] and fewer antiretroviral medications (ARVs) in one´s current regimen (2 or fewer different ARVs vs 3 or more different ARVs: AOR 17.0 (95% CI 3.7 – 97.6).

## Conclusions

Self-reported ART adherence in this sample of Estonian HIV-infected patients in clinical care was similar to rates observed in Western Europe and other developed countries. The results suggest that adherence education and support could be most beneficial, if specifically targeting the development of positive beliefs, reduction of negative expectations towards ART and when helping patients manage negative treatment experience, particularly with regimens including multiple ARVs.

